# GeneNetTools: tests for Gaussian graphical models with shrinkage

**DOI:** 10.1093/bioinformatics/btac657

**Published:** 2022-09-30

**Authors:** Victor Bernal, Venustiano Soancatl-Aguilar, Jonas Bulthuis, Victor Guryev, Peter Horvatovich, Marco Grzegorczyk

**Affiliations:** Center of Information Technology, University of Groningen, Groningen 9747 AJ, The Netherlands; Department of Mathematics, Bernoulli Institute, University of Groningen, Groningen 9747 AG, The Netherlands; Center of Information Technology, University of Groningen, Groningen 9747 AJ, The Netherlands; Center of Information Technology, University of Groningen, Groningen 9747 AJ, The Netherlands; European Research Institute for the Biology of Ageing, University Medical Center Groningen, University of Groningen, Groningen 9713 AV, The Netherlands; Department of Analytical Biochemistry, Groningen Research Institute of Pharmacy, University of Groningen, Groningen 9713 AV, The Netherlands; Department of Mathematics, Bernoulli Institute, University of Groningen, Groningen 9747 AG, The Netherlands

## Abstract

**Motivation:**

Gaussian graphical models (GGMs) are network representations of random variables (as nodes) and their partial correlations (as edges). GGMs overcome the challenges of high-dimensional data analysis by using shrinkage methodologies. Therefore, they have become useful to reconstruct gene regulatory networks from gene-expression profiles. However, it is often ignored that the partial correlations are ‘shrunk’ and that they cannot be compared/assessed directly. Therefore, accurate (differential) network analyses need to account for the number of variables, the sample size, and also the shrinkage value, otherwise, the analysis and its biological interpretation would turn biased. To date, there are no appropriate methods to account for these factors and address these issues.

**Results:**

We derive the statistical properties of the partial correlation obtained with the Ledoit–Wolf shrinkage. Our result provides a toolbox for (differential) network analyses as (i) confidence intervals, (ii) a test for zero partial correlation (null-effects) and (iii) a test to compare partial correlations. Our novel (parametric) methods account for the number of variables, the sample size and the shrinkage values. Additionally, they are computationally fast, simple to implement and require only basic statistical knowledge. Our simulations show that the novel tests perform better than DiffNetFDR—a recently published alternative—in terms of the trade-off between true and false positives. The methods are demonstrated on synthetic data and two gene-expression datasets from *Escherichia coli* and *Mus musculus*.

**Availability and implementation:**

The R package with the methods and the R script with the analysis are available in https://github.com/V-Bernal/GeneNetTools.

**Supplementary information:**

Supplementary data are available at *Bioinformatics* online.

## 1 Introduction

A Gaussian graphical model (GGM) (Edwards, 2000) consists of a network structure where random variables are nodes and their partial correlations are edges. Partial correlations are a full-conditional (linear) measure of the association between pairs of random variables, thus the GGM characterizes conditional independences.

To compute the partial correlations, it is necessary to standardize the inverse of the covariance matrix (i.e. the precision matrix). While the covariance matrix can always be estimated from data, in this case, the estimated matrix must be invertible and well-conditioned. This requirement ensures that the inverse of the covariance matrix exists and that its computation is stable (not damaged by numerical or estimation errors). For a dataset of *n* samples and *p* variables, the sample covariance estimator is invertible and well-conditioned only when *n* is greater than *p*. In other cases, it is invertible but ill-conditioned when *n* is comparable to *p*, or not even invertible when *n* is smaller than *p* (Ledoit and Wolf, 2004). These last two cases are common in large-scale applications, particularly in bioinformatics, where molecular information from a large set of genes are measured from few samples, such as transcripts, or proteins, and are often referred to as ‘high-dimensions’, ‘small n, large p’ or ‘n ≪ p’ scenarios.

Shrinkage, a type or regularization, deals with the ‘high-dimensional problem’ by stabilizing the estimator. Among the shrinkage approaches, we find Glasso (Friedman *et al.*, 2008) and the Ledoit–Wolf (LW) shrinkage (Ledoit and Wolf, 2003, 2004). Glasso estimates a sparse precision matrix with a *L*_1_ penalty to force some of its entries to be zero. The LW-shrinkage estimates an invertible covariance (or correlation) matrix using a bias towards a sparser matrix structure. These methodologies have made GGMs popular for large-scale applications, e.g. bioinformatics and biomedicine (Beerenwinkel *et al.*, 2007; Benedetti *et al.*, 2017; Das *et al.*, 2017; Imkamp *et al.*, 2019; Keller *et al.*, 2008; McNally *et al.*, 2015), where gene-expression alteration and (condition-specific) gene networks would reflect the underlying biological mechanisms (Barabási *et al.*, 2011).

However, one important challenge arises which is frequently ignored. These ‘shrunk’ partial correlations cannot be compared directly; different datasets imply distinct shrinkage values, and ultimately, different biases (Bernal *et al.*, 2019). For *L*_1_ penalty methods there are some methods to quantify pairs-wise changes in the precision and/or the partial correlation matrix (Liu, 2017; Yuan *et al.*, 2017; Zhang *et al.*, 2019), and consequently for differential network analysis (Class *et al.*, 2018; Zhang *et al.*, 2018, 2019). To the best of our knowledge, and despite its wide use, there is no approach for differential network analysis based on the LW-shrinkage GGM.

In this article, we adapt three classical (parametric) statistics to the LW-shrinkage (Schäfer and Strimmer, 2005). Our results provide a toolbox for (differential) network analysis that include (i) confidence intervals, (ii) a test for null effects and (iii) a test to compare ‘shrunk’ partial correlations. Each of these account for the number of variables, the sample size and shrinkage values.

## 2 Materials and methods

In this section, we present the LW shrinkage and GGMs (Ledoit and Wolf, 2003, 2004). We show how the shrinkage can be included in several test statistics. To this end, we will study a rescaled version of the partial correlation, which will prove to be easier to interpret and advantageous to develop statistical tests.

Throughout the manuscript, matrices are represented with uppercase bold letters, and estimators are denoted with a hat symbol (e.g. **X** is a matrix, and $\hat{X}$ is an estimator of X).

### 2.1 The ‘shrunk’ partial correlation

GGMs are network models where random variables are represented with nodes and partial correlations with edges. The partial correlation is a full-conditional correlation; it measures the linear association between two Gaussian variables, while all the others are held constant.

For a dataset of *p* variables and *n* samples, there are p(p-1)/2 partial correlations in total, which can be computed via

(1) $$P_{ij}=\frac{-\Omega_{ij}}{\sqrt{\Omega_{ii}\Omega_{jj}}},$$

where $P_{ij}$ denotes the partial correlation between the *i*-th and *j*-th variables, and Ω is the inverse of the *p*×*p* covariance matrix C (or equivalently, the inverse of the correlation matrix R).

The covariance matrix C can be estimated from data, e.g. with the sample covariance matrix ${\hat{C}}^{\mathrm{SM}}$, however, this task turns challenging when *n* is comparable to, or smaller than, *p*. The reason is that ${\hat{C}}^{\mathrm{SM}}$ becomes ill-conditioned (numerically unstable) or singular (non-invertible). In other words, the number of parameters to estimate (all the partial correlations) is too large relative to the amount of information available in the dataset (the sample size). This same issue arises with the sample correlation matrix ${\hat{R}}^{\mathrm{SM}}$. For instance, the Pearson’s correlation coefficient has degrees of freedom k = n -1, and the partial correlation coefficient k = n – (p-2) -1. In the well-conditioned case (n≥p+1), both degrees of freedom are positive. In the ill-conditioned case (n<p+1), for the partial correlation *k* would turn negative (and meaningless).

The LW shrinkage overcomes this issue via a ‘shrunk’ covariance matrix ${\hat{C}}^{\left[\lambda \right]}$ defined as,

(2) $${\hat{C}}^{\left[\lambda \right]}:=\left(1-\lambda \right){\hat{C}}^{\mathrm{SM}}+\lambda T.$$

Here λ ϵ (0, 1), also called the shrinkage value, represents the weight allocated to a target matrix T. The shrinkage λ has an optimal value that is obtained by minimizing the mean square error (Schäfer and Strimmer, 2005). In this work, T is taken as a diagonal matrix of variances, though other alternatives are possible. This choice shrinks the magnitudes of the covariances (off-diagonal), while the variances (diagonal) remain intact, which is equivalent to shrinking $R^{\mathrm{SM}}$ towards the identity matrix.

Using the inverse of ${\hat{C}}^{\left[\lambda \right]}$, denoted here by ${\hat{\Omega }}^{\left[\lambda \right]}$, Equation (1) becomes,

(3) $${{\hat{P}}^{\left[\lambda \right]}}_{ij}=\frac{-{{\hat{\Omega }}^{\left[\lambda \right]}}_{ij}}{\sqrt{{{\hat{\Omega }}^{\left[\lambda \right]}}_{ii}{{\hat{\Omega }}^{\left[\lambda \right]}}_{jj}}},$$

which is the ‘shrunk’ partial correlation between the *i*-th and *j*-th variables (Schäfer and Strimmer, 2005).

### 2.2 The distribution of the partial correlation—GeneNet

In the classical scenario, the Pearson’s correlation coefficient *r* and the partial correlation coefficient ρ follow the same probability distribution $f_{0}$, differing only in their degrees of freedom *k* (Fisher, 1924).

Under the null hypothesis $H_{0}:\rho =0$, the density of the partial correlation is

(4.1) $$f_{0}\left(\rho \right)=\frac{{\left(1-\rho^{2}\right)}^{(k-3)/2}}{\mathrm{Beta}\left(\frac{1}{2},\frac{k-1}{2}\right)},$$

with k=n-1-(p-2), which turns negative whenever n<p+1.

Equation (4.1) is used in the popular method GeneNet (Schäfer and Strimmer, 2005). This method computes ‘shrunk’ partial correlations with Equation (3) and (approximate) its *P*-values via empirical null-fitting. It relies in that Equation (4.1) is a reasonable approximation to the real ‘shrunk’ probability density, at least for small shrinkage values and sparse networks.

However, as analytical results about Equation (3) have been missing, appropriated discussions of effect size and significance have remained limited.

### 2.3 The distribution of the ‘shrunk’ partial correlation

In the ‘shrunk’ scenario, the same holds; the distribution of the ‘shrunk’ correlation $r^{\left[\lambda \right]}$ and ‘shrunk’ partial correlation $\rho^{\left[\lambda \right]}$ are the same, differing only in their degrees of freedom $k^{\left[\lambda \right]}$ (Bernal *et al.*, 2019).

Under the null hypothesis of $H_{0}:\rho^{\left[\lambda \right]}=0$, the density of the ‘shrunk’ partial correlation is

(4.2) $${f_{0}}^{\left[\lambda \right]}\left(\rho^{\left[\lambda \right]}\right)=\frac{{\left({\left(1-\lambda \right)\;}^{2}-{\rho^{\left[\lambda \right]}}^{2}\right)}^{(k^{\left[\lambda \right]}-3)/2}}{\mathrm{Beta}\left(\frac{1}{2},\frac{k^{\left[\lambda \right]}-1}{2}\right){\left(1-\lambda \right)\;}^{\left(k^{\left[\lambda \right]}-2\right)}},$$

where $k^{\left[\lambda \right]}$ has no closed form, but can be estimated via maximum likelihood.

At this point, we switch our attention to $y={\left(1-\lambda \right)}^{-1}\rho^{\left[\lambda \right]}$ which will prove convenient to derive some useful results. This transformation rescales the magnitude of $\rho^{\left[\lambda \right]}$, while the configuration space remains intact. The density in Equation (4.2) satisfies that

$$\int_{-\left(1-\lambda \right)}^{\left(1-\lambda \right)}{f_{0}}^{\left[\lambda \right]}\left(\rho^{\left[\lambda \right]}\right)d\rho^{\left[\lambda \right]}=1.$$

Replacing $\left(1-\lambda \right)\;y=\;\rho^{\left[\lambda \right]}$ and $\left(1-\lambda \right)\;dy={d\rho }^{\left[\lambda \right]}$ gives

$$\int_{-1}^{1}\left(\frac{{\left({\left(1-\lambda \right)\;}^{2}-{\left(\left(1-\lambda \right)\;y\right)}^{2}\right)}^{\frac{k^{\left[\lambda \right]}-3}{2}}}{\mathrm{Beta}\left(\frac{1}{2},\frac{k^{\left[\lambda \right]}-1}{2}\right){\left(1-\lambda \right)\;}^{\left(k^{\left[\lambda \right]}-2\right)}}\left(1-\lambda \right)\right)dy=1$$

and factoring the terms with $\left(1-\lambda \right)$, we have that

(4.3) $${f_{0}}^{\left[\lambda \right]}\left(\frac{\rho^{\left[\lambda \right]}}{\left(1-\lambda \right)}\right)=\frac{{\left(1-{\left(\frac{\rho^{\left[\lambda \right]}}{\left(1-\lambda \right)}\right)}^{2}\right)}^{\frac{k^{\left[\lambda \right]}-3}{2}}}{\mathrm{Beta}\left(\frac{1}{2},\frac{k^{\left[\lambda \right]}-1}{2}\right)}.$$

Which is equal to Equation (4.1). While $\rho^{\left[\lambda \right]}\;\epsilon \;\left[-\left(1-\lambda \right),\;\left(1-\lambda \right)\right]$, the rescaled version ${\left(1-\lambda \right)}^{-1}\rho^{\left[\lambda \right]}\;\epsilon \;\left[-1,\;-1\right]$.

In other words, ${\left(1-\lambda \right)}^{-1}\rho^{\left[\lambda \right]}$ is distributed as Pearson’s correlation with degrees of freedom $k^{\left[\lambda \right]}$. Therefore, methodologies developed for Pearson’s correlation can be readily adapted to the rescaled ‘shrunk’ partial correlation ${\left(1-\lambda \right)}^{-1}\rho^{\left[\lambda \right]}$.

### 2.4 Test for null-effects $H_{0}:\rho^{\left[\lambda \right]}=0$

Pearson’s and partial correlation coefficients are often tested using a *t*-statistic.

Let $\hat{\rho }$ be the estimated (partial) correlation from data, with population value $\rho_{\mathrm{true}}$ and degrees of freedom k. Then, under the null hypothesis of a zero-effect $H_{0}:\rho_{\mathrm{true}}=0$,

(5) $$t_{v\;=\;k-1}=\hat{\rho }\sqrt{\frac{k-1}{1-{\hat{\rho }}^{2}}},$$

follows a Student’s *t*-distribution with v = k -1. Equation (5) holds for Pearson’s and partial correlations each with their corresponding *k* (Cohen, 1988; Levy and Narula, 1978). An equivalent test for $\rho^{\left[\lambda \right]}$ is straightforward due to Equations (4.1)–(4.3).

Under the null hypothesis $H_{0}:{{\hat{\rho }}_{\mathrm{true}}}^{\left[\lambda \right]}=0,$

(6) $${t^{\left[\lambda \right]}}_{v\;=\;k^{\left[\lambda \right]}-1}=\frac{{\hat{\rho }}^{\left[\lambda \right]}}{\left(1-\lambda \right)}\sqrt{\frac{k^{\left[\lambda \right]}-1}{1-{\left(\frac{{\hat{\rho }}^{\left[\lambda \right]}}{\left(1-\lambda \right)}\right)}^{2}}},$$

which has a Student’s *t*-distribution with degrees of freedom $v=k^{\left[\lambda \right]}-$1, and tests the null hypothesis of a zero ‘shrunk’ partial correlation.

### 2.5 Confidence intervals of $\rho^{\left[\lambda \right]}$

Confidence intervals for Pearson’s (and partial) correlations are commonly computed using Fisher’s transformation $F\left(*\right)=\;\mathrm{arctanh}\left(*\right)$.

Let $\hat{\rho }$ be the estimated partial correlation with population value $\rho_{\mathrm{true}}$. Then, the Fisher-transformed $\hat{\rho }$, denoted here by $F\left(\hat{\rho }\right)$, is normally distributed with expectation $F\left(\rho_{\mathrm{true}}\right)$ and standard error $\sqrt{{\left(k-\;2\right)\;}^{-1}}$, or

(7) $$F\left(\hat{\rho }\right)∼\;N\left(F\left(\rho_{\mathrm{true}}\right),\;\frac{1\;}{\;k-\;2\;}\right).$$

Analogously, the Fisher’s transformed ‘shrunk’ partial correlation $F\left({\left(1-\lambda \right)}^{-1}{\hat{\rho }}^{\left[\lambda \right]}\right)$ is normally distributed with expectation $F\left({\left(1-\lambda \right)}^{-1}\rho_{\mathrm{true}}^{\left[\lambda \right]}\right)$ and standard error $\sqrt{\;{\left(k^{\left[\lambda \right]}-\;2\right)}^{-1}}$.

In other words,

(8.1) $$F\left(\frac{{\hat{\rho }}^{\left[\lambda \right]}}{\left(1-\lambda \right)}\right)∼\;N\left(F\left(\frac{\rho_{\mathrm{true}}^{\left[\lambda \right]}}{\left(1-\lambda \right)}\right),\;\frac{1\;}{\;k^{\left[\lambda \right]}-\;2\;}\right),$$

or

(8.2) $$\left(\frac{F\left(\frac{{\hat{\rho }}^{\left[\lambda \right]}}{\left(1-\lambda \right)}\right)-F\left(\frac{\rho_{\mathrm{true}}^{\left[\lambda \right]}}{\left(1-\lambda \right)}\right)}{\sqrt{\frac{1}{\left(k^{\left[\lambda \right]}-\;2\right)}}}\right)\;∼\;N\left(0,\;1\right).$$

Therefore, confidence limits for $\rho_{\mathrm{true}}^{\left[\lambda \right]}$ can be computed as follows. Let *z* ∼ N(0, 1) then,

$$P\left(-q_{\left(1-\frac{\alpha }{2}\right)}\leq z\leq q_{\left(1-\frac{\alpha }{2}\right)}\right)=\left(1-\frac{\alpha }{2}\right),$$

where $q_{\left(1-*\right)}$ is the $\left(1-*\right)$-th quantile of a normal random variable and the following inequality holds,

$$-q_{\left(1-\frac{\alpha }{2}\right)}\leq \frac{F\left(\frac{{\hat{\rho }}^{\left[\lambda \right]}}{\left(1-\lambda \right)}\right)-F\left(\frac{\rho_{\mathrm{true}}^{\left[\lambda \right]}}{\left(1-\lambda \right)}\right)}{\sqrt{\frac{1}{\left(k^{\left[\lambda \right]}-\;2\right)}}}\leq q_{\left(1-\frac{\alpha }{2}\right)}.$$

Both sides can be multiplied by $\sqrt{{\left(k-\;2\right)}^{-1}}$ and by minus one (which reverses the inequality), turning it into

$$F\left(\frac{{\hat{\rho }}^{\left[\lambda \right]}}{\left(1-\lambda \right)}\right)+\frac{q_{\left(1-\frac{\alpha }{2}\right)}}{\sqrt{\left(k^{\left[\lambda \right]}-\;2\right)}}\leq F\left(\frac{\rho_{\mathrm{true}}^{\left[\lambda \right]}}{\left(1-\lambda \right)}\right)\leq F\left(\frac{{\hat{\rho }}^{\left[\lambda \right]}}{\left(1-\lambda \right)}\right)-\frac{q_{\left(1-\frac{\alpha }{2}\right)}}{\sqrt{\left(k^{\left[\lambda \right]}-\;2\right)}}$$

applying the inverse transformation $F^{-1}\left(*\right)=\tanh\left(*\right)$, we have that

(9.1) $$P\left(\tanh\left(l^{\left[\lambda \right]}\right)\leq \frac{\rho_{\mathrm{true}}^{\left[\lambda \right]}}{\left(1-\lambda \right)}\leq \tanh\left(u^{\left[\lambda \right]}\right)\right)=\left(1-\frac{\alpha }{2}\right),$$

with

(9.2) $$\begin{array}{c} l^{\left[\lambda \right]}=F\left(\frac{{\hat{\rho }}^{\left[\lambda \right]}}{\left(1-\lambda \right)}\right)-\sqrt{\frac{1}{\left(k^{\left[\lambda \right]}-\;2\right)}}q_{\left(1-\frac{\alpha }{2}\right)}\\ u^{\left[\lambda \right]}=F\left(\frac{{\hat{\rho }}^{\left[\lambda \right]}}{\left(1-\lambda \right)}\right)+\sqrt{\frac{1}{\left(k^{\left[\lambda \right]}-\;2\right)}}q_{\left(1-\frac{\alpha }{2}\right)}. \end{array}$$

Equations (9.1) and (9.2) define the confidence intervals for the rescaled (population) ‘shrunk’ partial correlation ${\left(1-\lambda \right)}^{-1}\rho_{\mathrm{true}}^{\left[\lambda \right]}$.

Confidence intervals for the ‘shrunk’ partial correlation $\rho_{\mathrm{true}}^{\left[\lambda \right]}\;\epsilon \;[-\left(1-\lambda \right),\;\left(1-\lambda \right)]$ can also be obtained multiplying Equations (9.1) and (9.2) by $\left(1-\lambda \right)$. However, the rescaled version is superior in terms of its interpretability, as it is distributed as the classical Pearson’s correlation coefficient between −1 and 1.

### 2.6 Test to compare partial correlations $H_{0}:\rho^{\left[\lambda_{1}\right]}=\rho^{\left[\lambda_{2}\right]}$

Two (partial) correlation coefficients can be compared using Fisher’s transformation. The test is built by subtracting two Fisher-transformed coefficients [see Equation (7)] and assuming that their (unknown) population values $F\left(\rho_{\mathrm{true}}\right)$ are equal.

Two ‘shrunk’ (partial) correlation coefficients can be compared in the same way. Let us suppose that ${\hat{\rho }}^{\left[\lambda_{1}\right]}\;$and ${\hat{\rho }}^{\left[\lambda_{2}\right]}$ are estimates from two datasets with population values $\rho_{\mathrm{true}}^{\left[\lambda_{1}\right]}$ and $\rho_{\mathrm{true}}^{\left[\lambda_{2}\right]}$, and degrees of freedom $k^{\left[\lambda_{1}\right]}$ and $k^{\left[\lambda_{2}\right]}$. As discussed before, these estimates are not comparable *per se*, as they have different shrinkages—and scales—and a test for $H_{0}:\rho_{\mathrm{true}}^{\left[\lambda_{1}\right]}-\rho_{\mathrm{true}}^{\left[\lambda_{2}\right]}=0$ would be meaningless. However, their appropriately rescaled versions ${\left(1-\lambda_{1}\right)}^{-1}{\hat{\rho }}^{\left[\lambda_{1}\right]}$ and ${\left(1-\lambda_{2}\right)}^{-1}{\hat{\rho }}^{\left[\lambda_{2}\right]}$ are comparable.

Therefore, a test for $H_{0}:{\left(1-\lambda_{1}\right)}^{-1}\rho_{\mathrm{true}}^{\left[\lambda_{1}\right]}-\;{\left(1-\lambda_{2}\right)}^{-1}\rho_{\mathrm{true}}^{\left[\lambda_{2}\right]}=0$ is

(10) $$z^{\left[\lambda \right]}=\frac{F\left(\frac{{\hat{\rho }}^{\left[\lambda_{1}\right]}}{\left(1-\lambda_{1}\right)}\right)\;-\;F\left(\frac{{\hat{\rho }}^{\left[\lambda_{2}\right]}}{\left(1-\lambda_{2}\right)}\right)\;}{\sqrt{\;\frac{1\;}{\;\left(k^{\left[\lambda_{1}\right]}-\;2\;\right)}+\frac{1\;}{\;\left(k^{\left[\lambda_{2}\right]}-\;2\right)\;}}}∼\;N\left(0,\;1\right).$$

This *z*-statistics is distributed as N(0, 1). It tests whether two ‘shrunk’ partial correlations estimated from independent datasets are statistically different.

The practical implementation of the results in this section can be found in the Supplementary Material S1.

#### 2.6.1 Data

##### 2.6.1.1 Escherichia coli microarray data

This dataset consists of *E.coli* microarray gene-expression measurements. The study explores the temporal stress response upon expression of recombinant human superoxide dismutase (SOD) (Schmidt-Heck *et al.*, 2004), induced by isopropyl β-D-1-thiogalactopyranoside (a lactose analogue inducer of the lac operon). The measurements were collected at 8, 15, 22, 45, 68, 90, 150 and 180 min. In total, 102 out of 4289 protein-coding genes are differentially expressed across the nine time points. A log_2_-ratio transformation of transcript microarray intensity was applied with respect to the initial time points. The dataset was obtained from the R package *GeneNet* version 1.2.13. Accessed on April 7, 2022.

##### 2.6.1.2 Mus musculus RNA-sequencing data

Data are from single-end RNA-Seq reads from 21 male mice from two strains (B6, *n* = 10 and D2, *n* = 11). The dataset was downloaded from ReCount: http://bowtie-bio.sourceforge.net/recount/ under the PubMed Identifier 21455293 (Bottomly *et al.*, 2011), on April 7, 2022. Low expressed genes (<5 reads on average) were excluded from the data before pre-processing. In total, 223 genes out of 9431 are differentially expressed (adjusted by strain) with the R package limma at Benjamini–Hochberg adjusted false discovery rate <0.05 (Benjamini and Hochberg, 1995; Ritchie *et al.*, 2015). Before statistical analysis the transcript quantitative values were log_2_-transformed and upper quartile normalized.

## 3 Results

### 3.1 Analysis of simulated data

Here, we evaluate the performance of the proposed ‘shrunk’ *z*-test [Equation (10)] in terms of the area under the receiver operator curve (AUROC) and the area under the precision-recall curve (AUPRC). AUROCs and AUPRCs are metrics defined between 0 and 1 that measure the trade-off between true and false positives. We also compare our method against DiffNetFDR (Zhang *et al.*, 2019). DiffNetFDR is a computational approach for differential network analysis that uses the residuals obtained from lasso multivariate regression (Liu, 2017). The authors of DiffNetFDR recently reported a better performance compared to several other alternatives (Class *et al.*, 2018; Zhang *et al.*, 2018).

**Fig. 1.**
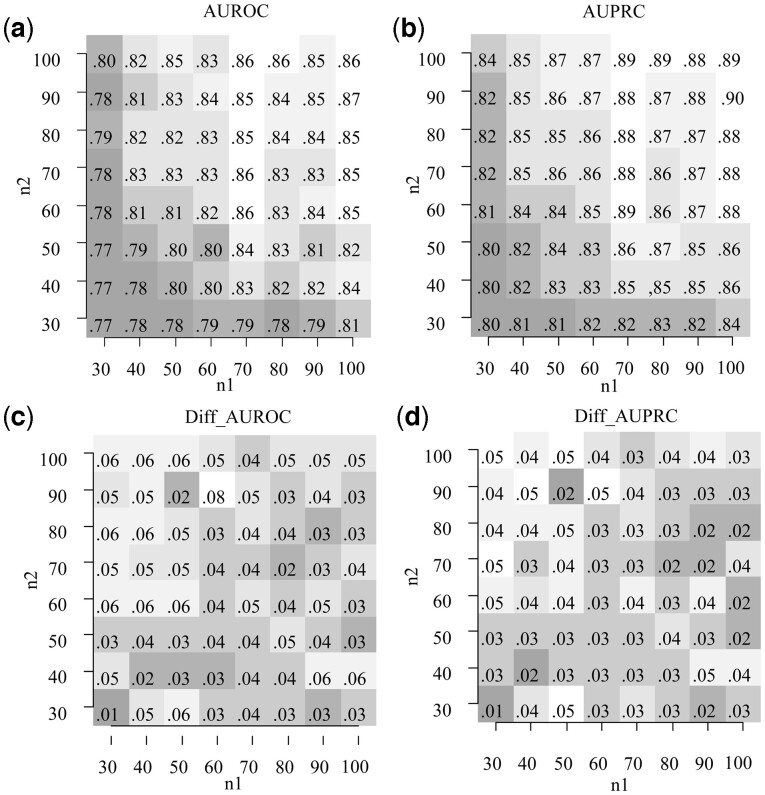
AUROC and AUPRC. (**a and b**) AUROCs and AUPRCs of the *z*-score [Equation (10)]. The performance increases with the sample size. (**c**) AUROCs of the *z*-score minus the AUROCs of DiffNetFDR. (**d**) AUPRCs of the *z*-score minus the AUPRCs of DiffNetFDR. Positive values show a better trade-off of true and false positives for the proposed *z*-statistics. Data were simulated from networks with *p* =100 nodes and sample sizes n1 and n2 between 30 and 100

We simulate data from one fixed network with 100 nodes and sample sizes 30, 40, 50,… and 100. For each pair of datasets, we reconstruct the partial correlations and compare them with the *z*-score [Equation (10)]. Figure 1a and b shows the AUROCs and AUPRCs varying the sample size. Figure 1c and d shows the difference in AUROCs and AUPRCs (in percentages) between the *z*-score [Equation (10)] and DiffNetFDR.

The proposed *z*-statistics shows higher AUROCs and AUPRCs (upper panels), and performs better than DiffNetFDR (lower panels). Supplementary Figures S1 and S2 show the AUROCs and AUPRCs for different networks sizes and for different proportion of edges.

### 3.2 Analysis of experimental data

#### 3.2.1 Effects of human SOD protein expression on transcript expression in *E.coli*

Here, we compare the *P*-values obtained with (i) the ‘shrunk’ *t*-test [Equation (6)] and (ii) the ‘shrunk’ probability density [Equation (4.2)]. Following previous works (Bernal *et al.*, 2019; Schäfer and Strimmer, 2005), the dataset is treated as static. The significance level is 0.05, and the optimal shrinkage is 0.18.

**Fig. 2.**
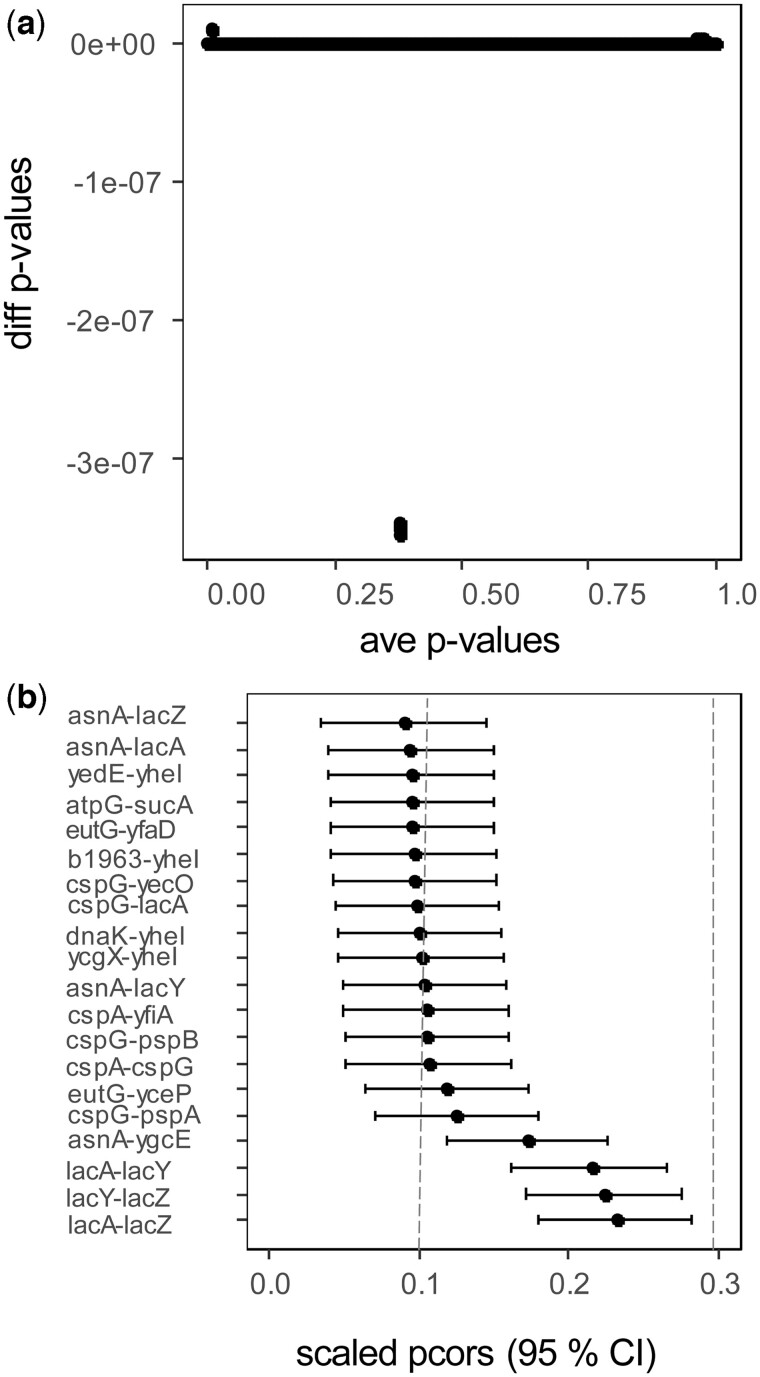
*Escherichia coli* microarray network analysis. (**a**) Bland–Altman plot between the *P*-values obtained from the *t*-test [Equation (6)] and the ‘shrunk’ probability density [Equation (4.2)]. The methods are equivalent as the differences are in the order 10^−7^. (**b**) Forest plot of partial correlations. The 15 strongest edges are displayed with their 95% confidence intervals. The vertical lines show the 0.1 and 0.3 thresholds for weak and mild correlations (Cohen, 1988)

The differences between the *P*-values are in the order 10^−07^. This is smaller than the tolerance value of the numerical integration (see previous method) and can thus be considered to be (numerically) zero. Both methods retrieved 238 edges in agreement previous analysis (Bernal *et al.*, 2019), though the ‘shrunk’ *t*-test was more than 10 times faster. The confidence intervals for the 15 strongest edges are displayed in Figure 2b. These include transcripts of lacA, lacY and lacZ genes, involved in the induction of the lac operon. The vertical lines show the 0.1 and 0.3 thresholds for weak and mild effects correlations (Cohen, 1988).

### 3.3 Analysis of *M.musculus* RNA-seq dataset

**Fig. 3.**
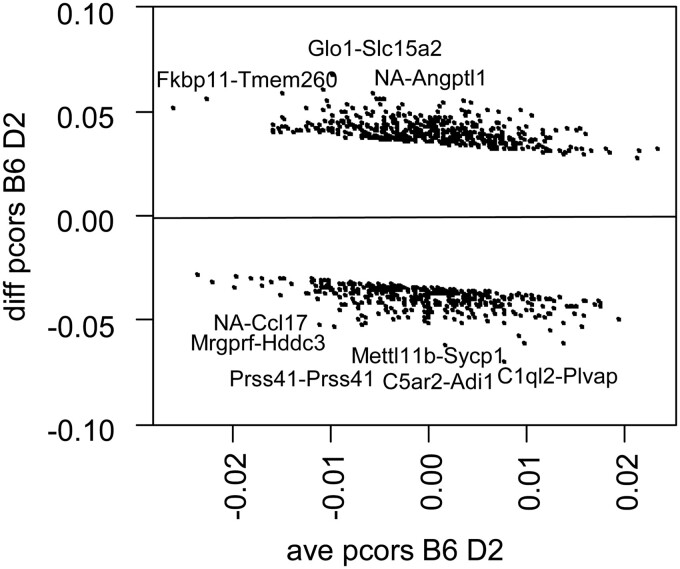
*Mus musculus* RNA-seq differential network analysis. Bland–Altman plot between the partial correlations for strains B6 and D2. The figure shows only the significantly different partial correlations at the 0.05 level (i.e. |*z*-score|> 1.96), and the names of the nine strongest gene pairs

Here, we compare the GGMs of two mice strains B6 and D2. Figure 3 presents the |*z*-score| that compares edge-wise the strains via Equation (10). The significance level is 0.05 (i.e. |*z*-score| >1.96), and the optimal shrinkages are 0.80 (B6) and 0.72 (D2).

In one case, 463 partial correlations (edges) are stronger in B6 than in D2, and relate to 193 genes. In the opposite case, 385 partial correlations are stronger in D2 than in B6, and relate to 184 genes. A recent B6-D2 comparison of the striatal proteome found 160 differentially expressed proteins, among which eight are well-known functional sequence variants at protein level (Parks *et al.*, 2019). The edges in our differential network analysis include four genes, which encode four of these eight proteins, namely; ALAD, GLO1, GABRA2 and COX7A2L.

## 4 Discussion

GGMs employ partial correlations to model interactions in the form of a network. The reconstruction of GGMs fall into the ‘high dimensional’ scenario in large-scale applications, which is a common case with gene-expression data. Shrinkage estimators solve the issues that arise in high dimensions, however, the ‘shrunk’ partial correlations depend on their shrinkage value, and cannot be compared directly.

In this work, we adapted some classical statistical tests to the partial correlations obtained with the LW shrinkage. We showed how an appropriate and simple transformation turns the ‘shrunk’ partial correlation into a (classical) Pearson’s correlation coefficient. Leveraging this fact, we derived (i) confidence intervals, (ii) a test for the hypothesis of a zero partial correlation and (iii) a test for the difference between pairs of partial correlations. These tests retrieve effect sizes (the strength and direction of the relationship), and *P*-values (the statistical significance), and account for the number of variables, the sample sizes and the shrinkage values.

Our simulations show that our test for differences between partial correlations (*z*-score) has an appropriate balance of false and true positives (Fig. 1a and b). Furthermore, its AUROC and AUPRC are higher than for the (recently published) state-of-the-art method DiffNetFDR (Fig. 1c and d). Our test of zero partial correlation (*t*-test) for the *E.coli* dataset is in agreement with previous results (Fig. 2a) (Bernal *et al.*, 2019), while being easier to implement and 10 times faster. Our differential network analysis (*z*-score) for the *M.musculus* dataset retrieves four well-known protein that might drive strain-specific regulation of gene expression (Parks *et al.*, 2019).

Finally, our methods are potentially useful to validate earlier network analyses, to compare gene regulatory networks from different experiments (differential networks analysis), and to further design of multi-omics/layer partial correlation methods. As most software includes efficient functions for a *t*-test, the arctanh and the quantiles of a normal density; our formulae are straightforward to implement, computationally fast even for large-scale applications and accessible to a broad audience with a basic background in Statistics.

## Supplementary Material

btac657_Supplementary_Data
