# Supplementary material for: GeneNetTools: tests for Gaussian graphical models with shrinkage
Source: Bioinformatics. 2022 Sep 30;38(22):5049–54. doi: 10.1093/bioinformatics/btac657 (PMC9665865; doi:10.1093/bioinformatics/btac657)
Supplement: btac657_Supplementary_Data [file btac657_supplementary_data.pdf]

Supplementary material for

GeneNetTools: Tests for Gaussian graphical models with shrinkage

Victor Bernal[1,2], Venustiano Soancatl-Aguilar[1], Jonas Bulthuis[1], Victor Guryev[4], Peter Horvatovich[3], Marco Grzegorczyk[2]

1 Center of Information Technology, University of Groningen, Groningen, 9747 AJ, The Netherlands. 2 Bernoulli Institute, University of Groningen, Groningen, 9747 AG, The Netherlands. 3 Department of Analytical Biochemistry, Groningen Research Institute of Pharmacy, University of Groningen, Groningen, 9713 AV, The Netherlands. 4 European Research Institute for the Biology of Ageing, University Medical Center Groningen, University of Groningen, Groningen, 9713 AV, The Netherlands.

Email: v.a.bernal.arzola@rug.nl

This document contains supporting material for the work *"GeneNetTools: Tests for Gaussian graphical models with shrinkage"*.

Contents

S1-Pseudo-code 1

S2-Figures 3

S1-Pseudo-code

In this subsection, the steps necessary to compute the statistics in Equation 6, 9, and 10 are presented. Let us consider two datasets D_1 and D_2 with p variables and sample sizes n_1 *and* n_2, respectively.

For dataset i:

1. Compute the 'shrunk' partial correlations $\rho^{[\lambda_i]}$
   a. Estimate the optimal shrinkage λ_i minimizing the Mean Square Error (Schäfer and Strimmer, 2005)
   b. Replace the output from 1.a in Equation 3 to obtain $\rho^{[\lambda_i]}$
2. Compute and store the following quantities
   a. Divide $\rho^{[\lambda_i]}$ by $(1-\lambda_1)$: $\frac{\rho^{[\lambda_i]}}{(1-\lambda_i)}$
   b. Apply Fisher's transformation: $F\left(\frac{\rho^{[\lambda_i]}}{(1-\lambda_i)}\right) = \text{arctanh}\left(\frac{\rho^{[\lambda_i]}}{(1-\lambda_i)}\right)$.
   c. Estimate the degrees of freedom $k^{[\lambda_i]}$ via Maximum Likelihood using Equation 4.3
3. Compute the p-value of $\rho^{[\lambda_i]}$
   a. Compute the t- statistic: $t^{[\lambda_i]}{}_{v=k^{[\lambda_i]}-1}$ using the outputs from 2.a-c in Equation 6
   b. Compute the p-value of $t^{[\lambda_i]}{}_{v=k^{[\lambda_i]}-1}$ (R built-in functions for the quantiles of a t-statistic with degrees of freedom $k^{[\lambda_i]}-1$)

4. Compute the confidence interval of $\rho^{[\lambda_i]}$

   a. Compute confidence intervals of $\rho^{[\lambda_i]}$ using the outputs from 2.a-c in Equation 9.2.

Repeat for the next dataset i + 1

5. Compare two partial correlations $\hat{\rho}^{[\lambda_1]}$ and $\hat{\rho}^{[\lambda_2]}$

   a. Compute the difference: $F\left(\frac{\hat{\rho}^{[\lambda_1]}}{(1-\lambda_1)}\right) - F\left(\frac{\hat{\rho}^{[\lambda_2]}}{(1-\lambda_2)}\right)$ using results from step 2 a-b

   b. Compute the standard error (SE) of the difference: $\sqrt{\frac{1}{k^{[\lambda_1]}-2} + \frac{1}{k^{[\lambda_2]}-2}}$ using results from step 2 c

   c. Compute the z-score $z^{[\lambda]}$ from Equation 10

   d. Compute the p-value of $z^{[\lambda]}$ (R built-in functions for the quantiles of a normal statistic)

End

S2-Figures

This section includes the supplementary Figures to the main manuscript.

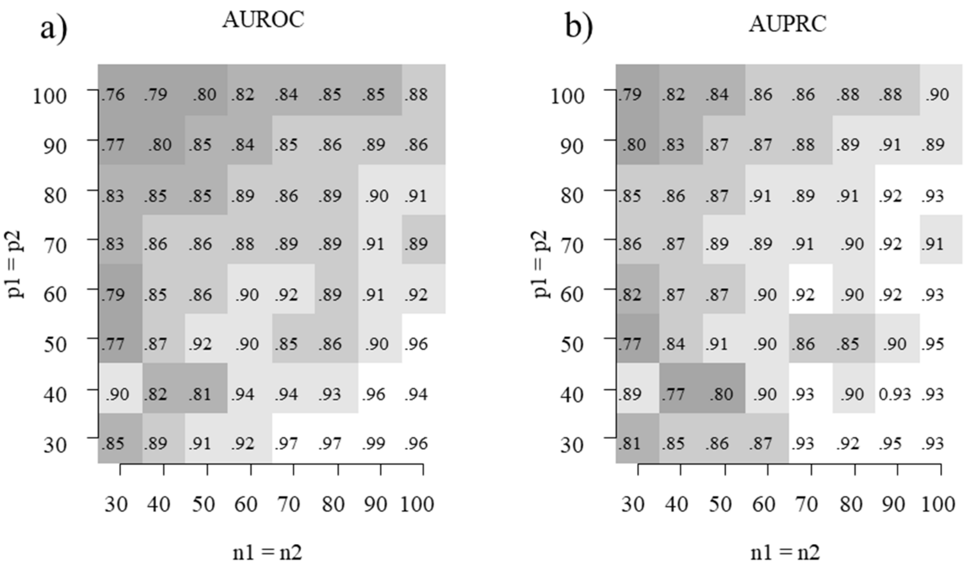

Figure S1. Areas under the receiver operator characteristic curve (AUROC) and the precision-recall curve (AUPRC) for different network sizes. The figure shows the AUROCs and AUPRCs of the z-score (Equation 10) for networks of sizes p1 = p2, and sample sizes n1 = n2, varying between 30 to 100. The proportion of edges in the (true) network is set to 3% (i.e. 148 edges). The network structure and the datasets were simulated with the R package GeneNet. It can be observed that for a given network size p, the performance increases with the sample size. As expected, for a given sample size n the performance decreases with the network size.

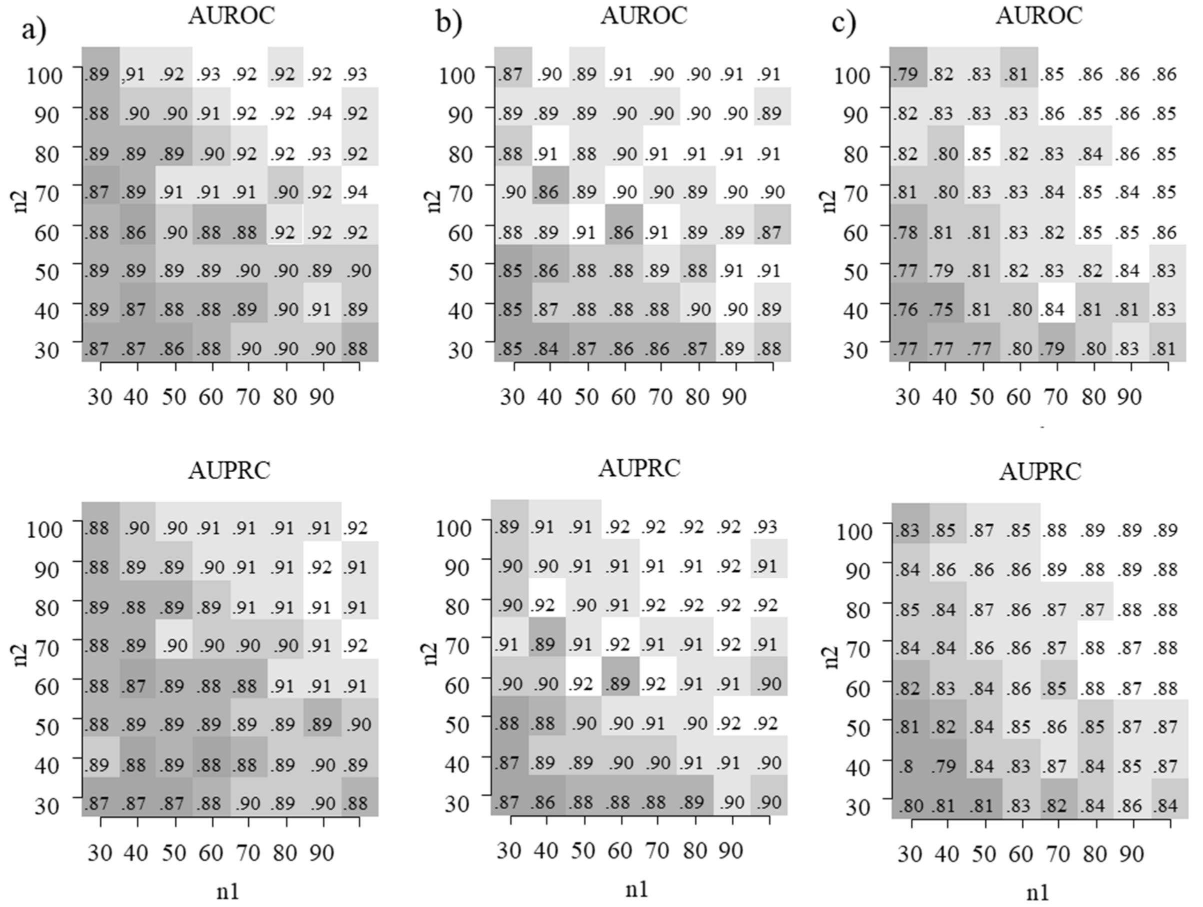

Figure S2. Area under the receiver operator characteristic curve (AUROC) and the precision-recall curve (AUPRC) for different number of edges. The figure shows AUROCs and AUPRCs of the z-score (Equation 10) for networks with different proportions of edges. Data were simulated from two network structures with size p1 = p2 = 100 nodes and with a sample size n1 = n2 varying between 30 and 100. In panels a, b, and c the proportion of edges of the (true) network are 1%, 2% and 3%. These proportion of edges correspond to 49, 99, and 148 edges, respectively. Network structures and data were simulated with the R package GeneNet. It can be observed that for a given network size p, the performance increases with the sample sizes.

References

Schäfer,J. and Strimmer,K. (2005) A shrinkage approach to large-scale covariance matrix estimation and implications for functional genomics. *Stat. Appl. Genet. Mol. Biol.*, **4**, 1175–1189.
